# Editorial: Bovine Tuberculosis—International Perspectives on Epidemiology and Management

**DOI:** 10.3389/fvets.2019.00202

**Published:** 2019-06-25

**Authors:** Andrew W. Byrne, Adrian R. Allen, Daniel J. O'Brien, Michele A. Miller

**Affiliations:** ^1^Veterinary Sciences Division, Agri-Food and Biosciences Institute, Belfast, United Kingdom; ^2^School of Biological Sciences, Queens University Belfast, Belfast, United Kingdom; ^3^Wildlife Disease Laboratory, Michigan Department of Natural Resources, Lansing, MI, United States; ^4^Faculty of Medicine and Health Sciences, Division of Molecular Biology and Human Genetics, DST-NRF Centre of Excellence for Biomedical TB Research, MRC Centre for Tuberculosis Research, Stellenbosch University, Cape Town, South Africa

**Keywords:** bovine tuberculosis, epidemiology, eradication, international, comparison

## Introduction

Bovine tuberculosis (bTB) remains a prominent zoonotic pathogen on the world stage, with significant impacts on animal and human health, and economic well-being. Eradication is hampered by a complex epidemiology, which in many countries involves wildlife hosts. Indeed, despite advances in understanding gleaned from national programs of bTB eradication, much of our understanding of transmission mechanisms, diagnostics, control, and multi-host infection systems remains opaque.

In this collection of *Frontiers in Veterinary Science*, as editors, we felt these limitations could best be addressed by adopting an international perspective. Localism understandably focuses on the fine details of problems at hand, but can perhaps overlook issues that only become apparent when compared to the experiences of others.

Below we summarize the papers published in this truly international collection, and highlight some themes. We trust readers will find these articles as stimulating to read as they were to edit.

### Theme: Modeling as a Tool for Understanding bTB

Modeling approaches were used to gain insights and make inferences on a number of different problems in bTB management.

Ladreyt et al. in their paper “*In silico* Comparison of Test-and-Cull Protocols for Bovine Tuberculosis Control in France,” developed a stochastic simulation model to explore the potential impacts of differing test-and-cull options relative to whole herd depopulations in terms of epidemiological effectiveness, cost and acceptability to stakeholders. The authors suggest the model will be of utility for decision support for comparing alternative control protocols.

In “Exploring the Fate of Cattle Herds With Inconclusive Reactors to the Tuberculin Skin Test,” Brunton et al. used statistical survival models to track the future risk of herds which retained inconclusive reactor (IR) animals. The authors reported significant increased future risk in herds with IR animals detected, relative to negative herds, and suggested that careful decision-making around the management of IR reactors needs to be employed to help mitigate such risk.

Statistical models were used by Frankena et al. in their paper “A New Model to Calibrate a Reference Standard for Bovine Tuberculin Purified Protein Derivative in the Target Species” to determine the potency of an in-house developed, reference standard of *M. bovis* purified protein derivative (PPDb). Secondarily, the model determined the precision and accuracy of the test relative to a standard (guinea pig) potency test. Such work will be important for ensuring uniformity of standards of PPD for bTB test diagnostics, as the Bovine International Standard (BIS) supply is limited.

Simulation and mathematical modeling has been a fundamental tool for bovine tuberculosis control, especially where wildlife reservoir species are involved. In “Modeling as a Decision Support Tool for Bovine TB Control Programs in Wildlife,” Smith and Delahay concentrate on the badger-TB episystem to highlight methodological approaches used to model disease dynamics and control interventions. The paper also highlights how future data collection could be integrated into modeling endeavors, and how such models could be optimally utilized.

### Theme: Host Genetics

The use of genetic selection to improve animal health has emerged from recent advances in genomics and their application to epidemiological data. Resistance to bTB is a heritable trait in cattle, and provides an additional tool by which the disease can be controlled.

In their Perspective article, “Can We Breed Cattle for Lower Bovine TB Infectivity?,” Tsairidou et al. pose a counterpoint to existing efforts to use genomic selection in cattle to improve resistance to bTB. Specifically, they raise the possibility that infectivity may be a separate genetic trait in cattle—indicating that some animals could be predisposed to being bTB “super-spreaders.” They also show by simulation modeling, how including selection for an infectivity phenotype alongside resistance could deliver reduced bTB prevalence for cattle industries.

In their Original Research article, “Impact of Genetic Selection for Increased Cattle Resistance to Bovine Tuberculosis on Disease Transmission Dynamics,” Raphaka et al. describe their development and evaluation of a simulation model which details the outcomes of varying intensities of genetic selection for bTB resistance in UK dairy cattle. Their findings show that adding genetic selection to bTB control strategies can aid in the reduction of bTB prevalence and severity of breakdowns. The authors suggest the use of genetic selection tools, alongside more traditional test and slaughter methods, could enhance eradication schemes.

### Theme: Pathogen Genetics

Genomic methods are revolutionizing traditional molecular epidemiological approaches to disease source attribution, principally due to their much superior resolution. Application to bTB infectious systems promises to improve our knowledge of transmission dynamics.

Orloski et al. provide a current description of the diversity of *M. bovis* isolates in cattle and farmed cervids in “Whole Genome Sequencing of *Mycobacterium bovis* Isolated from Livestock in the United States, 1989-2018.” The authors conclude that the use of whole genome sequencing (WGS) can reduce time and costs associated with epidemiological investigations, provides a powerful tool for advancing our understanding of transmission, and may improve eradication programs.

Similarly, in “Whole Genome Sequencing for Determining the Source of Mycobacterium bovis Infections in Livestock Herds and Wildlife in New Zealand,” Price-Carter et al. demonstrated the increased resolution of WGS for identifying sources of infection in outbreaks, especially in areas complicated by wildlife reservoirs. WGS was also able to provide information on the evolution of *M. bovis* within New Zealand animal populations, becoming a key component in their eradication strategy.

### Theme: Wildlife Reservoirs

In multi-host epidemics, control or eradication of bTB in domestic hosts is often unachievable if disease control in wildlife reservoir populations is not simultaneously implemented.

As highlighted by both Buddle et al. and Gormley and Corner there are few options available to control TB in wild populations that are both effective and socially acceptable. Buddle et al. in “Efficacy and Safety of BCG Vaccine for Control of Tuberculosis in Domestic Livestock and Wildlife,” highlight Bacillus Calmette Guerin (BCG) vaccination of wildlife as one such approach. The authors highlight the evidence to suggest BCG can induce protection in European badgers, white-tailed deer, wild boar, and brushtail possums. Gormley and Corner explored the palatability of wildlife interventions to different stakeholders and value systems in their review “Wild Animal Tuberculosis: Stakeholder Value Systems and Management of Disease.” The authors suggest that factors influencing consensus on management approach can depend on the species in question, the economic cost-benefit and ethical considerations. The review identified that interventions that are acceptable in one region, may not be agreed upon in another region, even amongst broadly similar stakeholder groups.

Vaccination with BCG has been shown to reduce disease in humans caused by *M. tuberculosis*, and Palmer and Thacker have also recently shown its potential for wildlife in “Use of the human vaccine, *Mycobacterium bovis* Bacillus Calmette Guerin in deer.” Decreased disease severity in vaccinated deer would likely be accompanied by decreased disease transmission. Progress on the development of oral baits for vaccines will facilitate the effort to implement this potentially valuable tool for addressing *M. bovis* infection in deer, and subsequent spread to livestock.

Bouchez-Zacria et al. in “The Distribution of Bovine Tuberculosis in Cattle Farms Is Linked to Cattle Trade and Badger-Mediated Contact Networks in South-Western France, 2007–2015,” used a network modeling approach to investigate cattle farms' bTB risk in France, utilizing both cattle trade data in concert with badger contact networks based on inferred badger home ranges, and *M. bovis* molecular typing. This work highlighted how both spatial relationships and trade relationships between farms, along with linkages associated with badger territorial behavior can be attributed to bTB risk, highlighting the complexity of multi-host epidemics.

Human-caused aggregations of animals are a challenging tuberculosis management problem. In “Baiting and Feeding Revisited: Modeling Factors Influencing Transmission of Tuberculosis Among Deer and to Cattle,” Cosgrove et al. extend previous modeling work to investigate how spatial and temporal persistence, density and attractiveness of feeding sites for wild deer affect both prevalence in deer and subsequent interspecies transmission. They show why feeding of deer is a relevant issue not only for hunters and wildlife managers, but for cattle producers and agriculture agencies as well.

The essential role of on-farm biosecurity in managing bovine tuberculosis is one of the focal points of VerCauteren et al's. “Persistent Spillback of Bovine Tuberculosis From White-Tailed Deer to Cattle in Michigan, USA: Status, Strategies, and Needs.” The authors provide a much-needed summary of biosecurity research undertaken in Michigan to date, and lessons learned. In addition, they make a case for management tools as yet un-, or at least under-utilized: vaccination of wild deer, precision culling of deer on farms, and, notably, strategic habitat manipulations to spatially redistribute deer.

Also focusing on white tailed deer in the USA, Cross et al. “Bovine Tuberculosis Management in Northwest Minnesota and Implications of the Risk Information Seeking and Processing (RISP) Model for Wildlife Disease Management” focuses on how deer hunters sought and acquired information on potential human health risks stemming from *M. bovis* exposure via hunting. Understanding how stakeholders obtain knowledge and form perceptions is crucial to implementing disease management in which they are necessary participants.

A pair of studies in this Research Topic addresses reservoirs of *Mycobacteria* in European wildlife. In “Mycobacterium caprae Infection of Red Deer in Western Austria–Optimized Use of Pathology Data to Infer Infection Dynamics*,”* Nigsch et al. provide for the first time in English a detailed description of the *M. caprae* outbreak spilling over to sympatric pastured cattle. They advocate for the use of a summary measure of detailed pathology findings—the Patho Score—as a quick and easy means of drawing broader inferences about the epidemiology of the disease in red deer, with implications for control in cattle.

In France, Réveillaud et al. recount the design and implementation of a nationwide system for *M. bovis* testing. “Infection of Wildlife by *Mycobacterium bovis* in France Assessment Through a National Surveillance System, Sylvatub” details the structure and integration of a surveillance system that acquires samples from diverse governmental and non-governmental partners, via both active and passive means. Thus, far, in addition to cattle, infection has been detected mainly in badgers and wild boar, although not to the extent noted elsewhere in Europe.

Many countries where *M. bovis* cycles between cattle and free-ranging wildlife declare the necessity of eliminating the disease, but such goals are typically easier proclaimed than achieved. Exactly how can eradication be methodically pursued in the face of real-world logistical, cost and policy constraints? In “Roll-Back Eradication of Bovine Tuberculosis (TB) From Wildlife in New Zealand: Concepts, Evolving Approaches, and Progress,” Nugent et al. characterize a Bayesian “Proof of Freedom” framework currently being implemented on an area-specific basis, as well as how, incorporating decision theory, the approach is being scaled up to the national level.

### Theme: The Importance of Diagnostics

As well as the diagnostic regent variation (see Frankena et al.), host physiological and immunological status can affect the performance of diagnostic tests. Kelly et al. in “Association of Fasciola gigantica Co-infection with Bovine Tuberculosis Infection and Diagnosis in a Naturally Infected Cattle Population in Africa” present evidence to suggest an association between a liver fluke species' infection and bTB risk in different cattle breeds in Cameroon. Parasite co-infection is believed to affect the balance between Th1 and Th2 arms of the immunological response to infection, potentially impacting either diagnosis or disease progression. Kelly et al. report a complex interaction between the presence of bTB lesions and the performance of the interferon-gamma ante-mortem test in mixed breed animals, but not Fulani cattle.

In “Validation of a Real-Time PCR for the Detection of Mycobacterium tuberculosis Complex Members in Bovine Tissue Samples,” Lorente-Leal et al. discuss their development and validation of a real time PCR assay for the detection of members of the *Mycobacterium tuberculosis* complex (MTBC) organisms in bovine tissues. Their assay exhibits good sensitivity and specificity relative to culture, and whilst the latter remains the gold standard for disease confirmation, the speed of the PCR assay could rapidly increase turnaround times, which is potentially of substantial epidemiological importance.

Hadi et al. describe in “Development of a Multidimensional Proteomic Approach to Detect Circulating Immune Complexes in Cattle Experimentally Infected with *Mycobacterium bovis*,” the preliminary findings of a novel proteomic method for the diagnosis of bTB infection in cattle. They identify immune complex proteins from the MTBC in experimentally infected cattle suggesting potential for this type of diagnostic approach. They also identify ways in which the method can be improved before validation on a larger dataset.

Effective management of bTB requires development of accurate diagnostic tests in the wide spectrum of susceptible hosts. However, the conventional tuberculin skin test has low sensitivity in camelid species. In “Development and Evaluation of a Serological Assay for the Diagnosis of Tuberculosis in Alpacas and Llamas,” Infantes-Lorenzo et al. describe the development of an ELISA based on the antigen P22 that had high specificity and sensitivity. This provides improved methods of detecting *M. bovis* and *M. microti* in New World camelids.

### Theme: “The Human Component”

The “human component” of bTB epidemiology and control was highlighted in a number of papers relating to societal values and ethics of bTB control, as well as human zoonotic risk.

In “Risk Perceptions and Protective Behaviors Toward Bovine Tuberculosis Among Abattoir and Butcher Workers in Ethiopia,” Fekadu et al. describe the findings of their modeling study elucidating the risk factors associated with consumption of raw meat, and contraction of bTB by abattoir workers in Ethiopia. The data showed that despite workers' knowledge of the dangers involved in eating raw meat, and awareness of bTB risk, there was little uptake of preventative behavior, particularly in male workers and older demographics. The authors recommend tailoring public health interventions toward not only increasing awareness of zoonotic risk, but also influencing behavioral change.

In “TB Control in Humans and Animals in South Africa: A Perspective on Problems and Successes,” Meiring et al. draw from the lessons of human TB control in South Africa and provide a comparison of these steps to bTB. Reduction in human TB incidence in this high burden country has been achieved through antimicrobial treatment and increasing awareness, both of which are lacking for bTB. Lack of effective movement controls, mandatory testing program, veterinary resources including state diagnostic laboratories, point-of-care tests for cattle, and presence of bTB in wildlife populations contributes to persistence of disease. A multi-sector approach, as seen for human TB, is needed to address bTB.

Although bTB is a global disease, it can be neglected in smaller nations. Borja et al. describe findings of bTB in Fiji in “A Retrospective Study on Bovine Tuberculosis in Cattle on Fiji: Study Findings and Stakeholder Responses.” Despite a bTB control program, goals were not being achieved due to lack of training of staff conducting TB testing, absence of standard data collection and unregulated movement of cattle. Revision of the program resulted in increased detection but also farmers' concerns. This study highlights the technical and social challenges to effective disease control.

Perceptions of cattle farmers are critical to implementing tuberculosis management strategies that ultimately rely upon their proactive cooperation and compliance. In “Negotiated Management Strategies for Bovine Tuberculosis: Enhancing Risk Mitigation in Michigan and the UK,” Little compares experiences with cattle producers in the two countries facing the need for heightened biosecurity on their farms. Rather than relying solely upon voluntary compliance, she demonstrates the value of negotiated outcomes in obtaining producer support for more stringent regulations necessitated by the presence of *M. bovis* in wildlife.

Finally, in their Hypothesis and Theory article, “Bovine Tuberculosis in Britain and Ireland – A Perfect Storm? The Confluence of Potential Ecological and Epidemiological Impediments to Controlling a Chronic Infectious Disease,” Allen et al. focus on the ongoing problems of eradicating bTB in Britain and Ireland—asking, why are these territories so different from their continental European neighbors? Is there a cocktail of unique epidemiological and ecological factors that are hindering efforts? If so, what could they be, and how can they be addressed? Suggested factors included the presence of a wildlife reservoir, differing diagnostic approaches, variation in pathogen genetics, co-infection, the structure and density of animal movement trade networks, and the potential for environmental persistence and a benign climate.

## Conclusion

**Figure 1:**
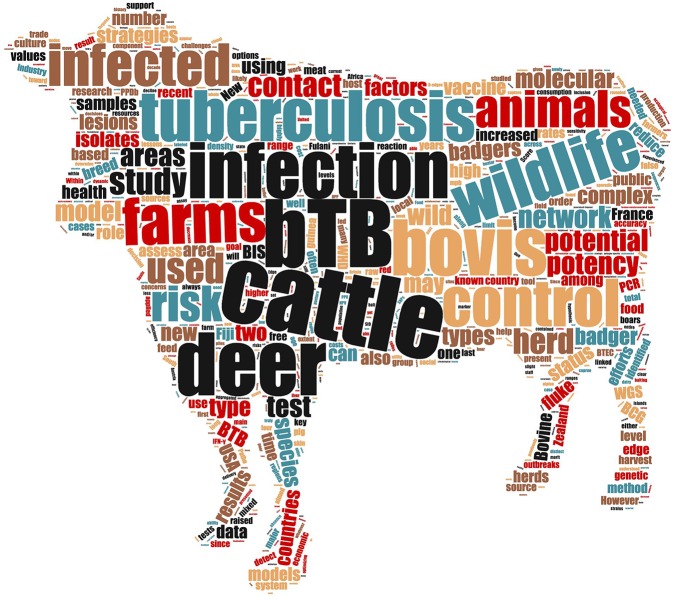
Frequency weighted “word cloud” using abstracts from the Research Topic “Bovine tuberculosis—international perspectives on epidemiology and management.”

This Research Topic was contributed to by 150 authors, representing over 15 countries. As highlighted, some common themes emerged from these contributions (Figure 1). The value, utility, and contribution to understanding bTB made from different modeling approaches was prominent. Wildlife was a significant component of the epidemiology of bTB in a number of different countries (Ireland, the UK, France, USA, South Africa, and New Zealand), with disease risk management technically and sociologically challenging. Genetic tools were found to be a core enabler to derive new insights into bTB epidemiology at both the host and pathogen levels. The importance of vaccines and diagnostics, their performance characteristics, and their standardization remains highly relevant. Finally, the “human component” was a significant theme, in terms of societal values, ethics and perceived (and realized) cost-benefits of interventions, as well as the practical risk of *M. bovis* infection as a zoonotic pathogen. These themes reflect the reality that bTB control across countries is a multi-factorial problem requiring significant specialist input from various disciplines, in conjunction with “buy-in” from stakeholders across broader society, in order to move efforts from control to eradication.

## Author Contributions

All authors listed have made a substantial, direct and intellectual contribution to the work, and approved it for publication.

### Conflict of Interest Statement

The authors declare that the research was conducted in the absence of any commercial or financial relationships that could be construed as a potential conflict of interest.

